# Dichotic listening performance and interhemispheric integration after stress exposure

**DOI:** 10.1038/s41598-020-77708-5

**Published:** 2020-11-30

**Authors:** Gesa Berretz, Julian Packheiser, Oliver T. Wolf, Sebastian Ocklenburg

**Affiliations:** 1 Department of Biopsychology, Institute of Cognitive Neuroscience, Faculty of Psychology, Ruhr University Bochum, Bochum, Germany; 2 Department of Cognitive Psychology, Institute of Cognitive Neuroscience, Faculty of Psychology, Ruhr University Bochum, Bochum, Germany; 3 Department of Psychology, University of Duisburg-Essen, Essen, Germany

**Keywords:** Psychology, Neuroendocrinology, Neuroscience, Stress and resilience

## Abstract

Functional hemispheric asymmetries (FHAs) have been thought to be relatively stable over time. However, past research has shown that FHAs are more plastic than initially thought. Endocrinological processes have been demonstrated to alter FHAs. As the product of the stress-activated hypothalamus–pituitary–adrenal axis, cortisol influences information processing at every level from stimulus perception to decision making and action. To investigate the influence of acute stress on FHAs, 60 participants performed a Banich–Belger task, as well as a verbal and an emotional dichotic listening task in two sessions. One session included a stress induction via the Trier Social Stress Test, the other session included a control procedure. We calculated across-field advantages (AFAs) in the Banich–Belger task and lateralization quotients for reaction times and responses per side in both dichotic listening tasks. There were no significant differences between the stress and control session in the dichotic listening tasks. In contrast, there was evidence for an influence of cortisol and sympathetic activation indicated by salivary alpha amylase changes on AFAs in the Banich–Belger task. This indicates that acute stress and the related increase in cortisol do not influence dichotic listening performance. However, stress does seem to affect interhemispheric integration of information. Future research using EEG, fMRI and pharmacological interventions is needed to further characterize the relation of hemispheric asymmetries and acute stress.

## Introduction

Although the brain seems to be symmetrical at first glance, it is organized in a fundamentally asymmetrical way^1^. These brain asymmetries are observable not only on the micro- and macrostructural level, but on the functional level as well^2^. Functional hemispheric asymmetries (FHAs) emerge as both left- and right-hemispheric networks contribute to task processing with each hemisphere being specialized and dominant for different aspects of the task.

Clinical research has indicated that some neurodevelopmental and mental disorders are accompanied by changes in asymmetry patterns (e.g. autism^3^, dyslexia^4^, schizophrenia^5^). Many of these disorders are also associated with alterations in the patient’s physiological stress response. The body is equipped with two systems that respond to acute stress. The faster acting system is the sympathetic nervous system triggering release of adrenaline and noradrenaline from the adrenal medulla. The slower acting Hypothalamus–Pituitary–Adrenal (HPA) axis releases corticotrophin-releasing hormone from the hypothalamus to stimulate secretion of adrenocorticotropic hormone from the anterior pituitary, which in turn leads to release of cortisol from the adrenal medulla^6,7^. Acute stress has been shown to influence the entire information processing stream: it affects vigilance and attention^8,9^, memory processes^10,11^ and executive functioning^12^.

In a recent review, we proposed a model in which early life stress as well as chronic stress not only increases the risk for mental and neurodevelopmental disorders but also changes structural and functional hemispheric asymmetries leading to the aberrant lateralization patterns seen in these disorders^13^. Also in the non-clinical population, early life stress has been associated with changes in asymmetries. For example, low birth weight as a marker of less beneficial intrauterine development has been associated with decreased right-handedness^14^. Moreover, many mental and neurodevelopmental disorders display changes in cortisol with higher cortisol levels being observed in individuals with for example schizophrenia and depression^15,16^. Contrary to this, patients with PTSD have been shown to display an increase in verbal hemispheric asymmetries^17^. This raises the question whether not only long-term alterations in cortisol due to chronic or early life but also temporary changes in cortisol due to acute stress can induce similar effects.

The effects of acute stress on FHAs however are not well understood. A preliminary study by Brüne et al.^18^ used a visual half field paradigm to investigate the influence of acute stress on FHAs. The authors reported that participants responded faster to negative stimuli in the left visual half field and to positive stimuli in the right visual half field in the stress condition. No such effects were found in the control condition indicating that acute stress induced a functional asymmetry in emotional processing. Moreover, stress has been shown to induce right-hemispheric activation in subjects who score high in hostility and enhance right-hemispheric performance^19,20^.

The effects of stress on FHAs could be mediated by two possible mechanisms: in the affective model, the negative affect accompanying acute stress could differentially affect both hemispheres. One of the dominant theories on emotional brain lateralization is the right-hemisphere hypothesis which proposes that the right hemisphere is more dominant for processing of positive and negative emotions^21,22^. Regarding negative emotions, the valence hypothesis similarly suggests a right hemispheric processing dominance^23^. It could be speculated that the right hemisphere is selectively primed by stress resulting in changes in FHAs. On the other hand, the effects of stress on FHAs could be mediated by its influence on the corpus callosum. In the psychoneuroendocrine model^24^, stress related cortisol release could influence FHAs by influencing glutamatergic and GABAergic neurotransmission via the corpus callosum^25,26^: Cortisol release in response to acute stress increases glutamatergic transmission and thus could also increase FHAs.

The aim of the current study was to investigate the influence of acute stress related changes in cortisol on FHAs. For that purpose, participants performed three different tasks assessing FHAs and interhemispheric integration. In the verbal dichotic listening task, different syllables are simultaneously presented to the left and right ear. Participants with typical left hemispheric lateralization for language report more syllables from the right ear^27,28^. In the emotional dichotic listening task, the same syllable in different emotional intonations is simultaneously presented to the left and right ear. Participants with typical right hemispheric lateralization for emotional processing report more syllables from the left ear^29,30^. Both dichotic listening tasks examine examples of hemispheric lateralization, namely in the language and emotion processing domain. This lateralization depends among other factor on interhemispheric inhibition of the non-dominant by the dominant hemisphere via the corpus callosum.

The Banich–Belger task measures interhemispheric integration by asking participants to compare stimuli in the right and left visual half field in two levels of difficulty. In the more difficult condition, participants typically show an advantage of integrating information across both hemispheres compared to one hemisphere alone. Thus, this task examines the integration of information across both hemispheres, which is dependent on information transfer across the corpus callosum^31^.

We expect that, on average, participants will show a right-ear advantage indicating left-hemispheric processing in the verbal dichotic listening task and a left-ear advantage indicating right-hemispheric processing in the emotional dichotic listening task in accordance with previous studies. We also expect shorter reaction times during across field trails in the more difficult name matching condition in the Banich–Belger task.

In line with the psychoneuroendocrine hypothesis of stress on FHAs, we predict increased cortisol due to acute stress increases interhemispheric transmission and shorten transfer times in the Banich–Belger task. Since stress should increase inhibition by the dominant hemisphere, in both verbal and emotional dichotic listening tasks stronger FHAs could be expected compared to the non-stressful control session. Further, we propose that participants with a stronger cortisol response to the TSST show larger asymmetries in hemispheric processing of language and emotional stimuli.

## Methods

### Participants

Participants were recruited at the Ruhr University Bochum, Germany. To determine the sample size, we performed a power analysis using g*Power^32^. We used an α-error probability of 0.05 and a power of 0.95. Based on the data by Brüne et al.^18^ we estimated the effect size to be small (partial η^2^ = 0.07). This resulted in an overall sample size of 53. To account for possible technical problems during data collections, we included slightly more participants in the final sample size. The sample consisted of 60 participants (29 women and 31 men) ranging in age from 18 to 36 (mean = 24.75, SD = 3.75). Only one participant had a negative handedness LQ indicating strong left-handedness as measured with the Edinburgh Handedness Inventory^33^. All other participants had positive handedness LQs indicating right-handedness (M = 83.83, SD = 30.49, min =  − 100, max = 100). All participants had no history of mental or neurological disorders; all were non-smokers and had no prior experience with the stress induction paradigm. Participants were screened with an audiometer to assure unimpaired hearing ability (cutoff: 15 dB difference between ears). To control for possible influences on cortisol and response during the experiment, all participants had a body mass index between 18.5 and 25 kg/m^2^, took no medication, drugs or hormonal contraception and did not work in shift work^34,35^. The study was approved by the local ethics committee of the Faculty of Psychology at the Ruhr University Bochum. All participants gave their written informed consent and were treated in accordance with the Declaration of Helsinki.

### Procedure

Participants were invited for two test sessions. Sessions took place between 2 and 6 pm to control for circadian changes in cortisol. Women were only tested in the luteal phase of their cycle three to eight days after onset of their period to control for hormonal changes possibly affecting FHAs^36^. After completion of baseline subjective stress and cortisol measurements, participants began the stress induction or control procedure. For stress induction, we used the Trier Social Stress Test (TSST)^37^. After a five-minute preparation period, participants have to give a five-minute oral presentation about their positive traits in a mock job interview followed by a mental arithmetic task (subtracting in steps of 17) for a total of 10 min. During the presentation and the arithmetic task, a panel consisting of a woman and a man dressed in lab coats evaluates the participants. The panel refrains from giving any positive feedback. Further, the participant’s face is videotaped and the video is streamed to a nearby monitor so that participants can see their own performance.

As a control procedure that does not induce stress, we utilized the Placebo-TSST (P-TSST^38^). The P-TSST also consists of a preparation period, an oral presentation and an arithmetic task. However, participants are neither monitored nor filmed, and the mental arithmetic task is less taxing (counting foreword in steps of 15). As the P-TSST lacks the stressful elements of the TSST like social evaluation and pressure to perform^39^ while mimicking its task demands, it is a suitable control procedure. The order of TSST and P-TSST sessions were pseudo-randomized so that half the participants performed the TSST in the first session and the P-TSST in the second session and vice versa. Participants were re-invited for the second session between one to seven days following the first session.

In both sessions, participants completed three different tasks. The order in which the paradigms were conducted was pseudo-randomized within each session and across participants. All paradigms were programmed and presented using the software Presentation (Neurobehavioral Systems, Inc., Albany, USA).

**Figure 1:**
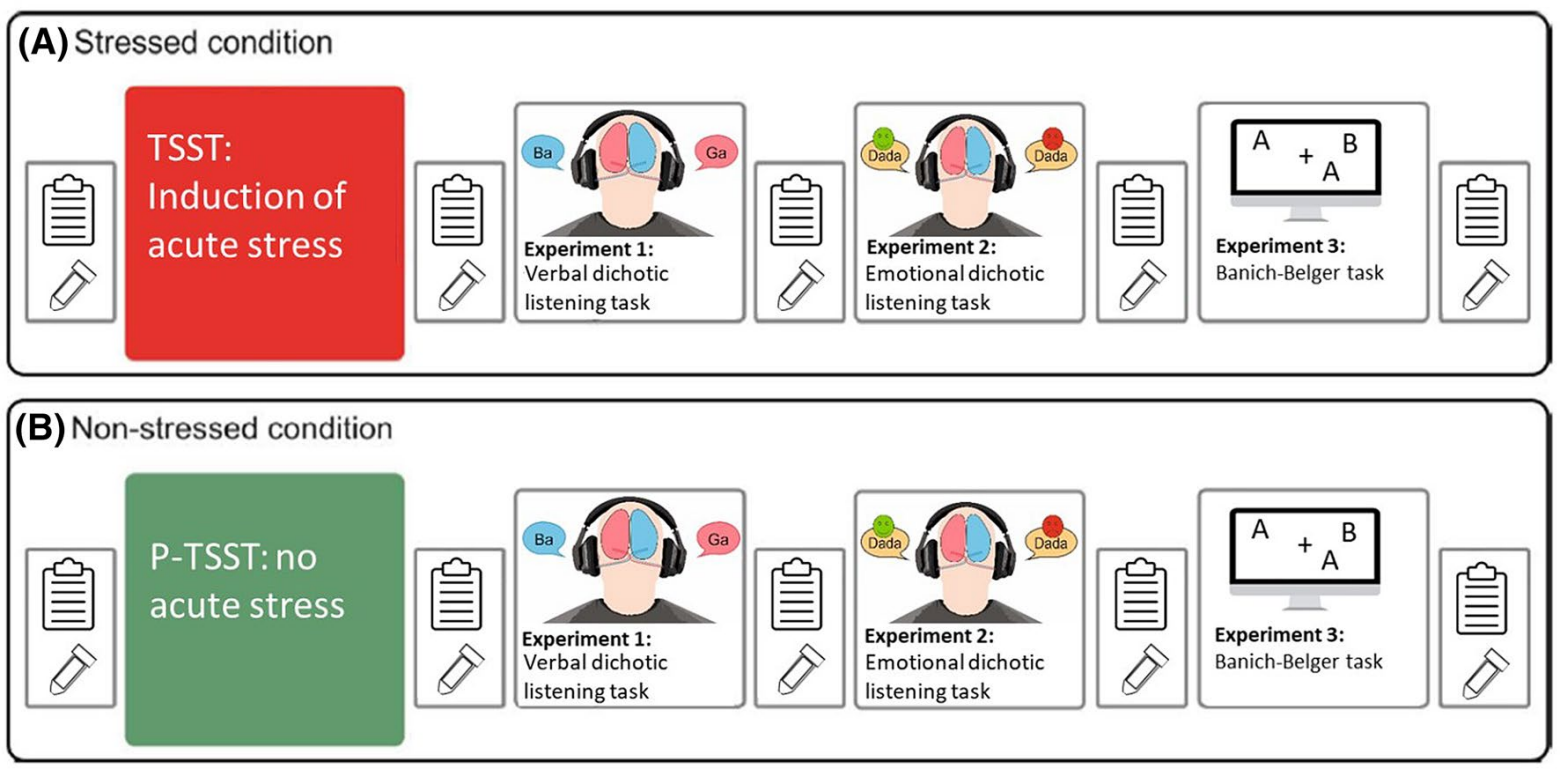
Experimental design. After TSST or P-TSST, the participant completes three experimental tasks to measure functional hemispheric asymmetries. Before the stress induction and after each section of the experiment, cortisol and affect are assessed. (**A**) In the stress session, participants undergo the TSST. (**B**) In the control session, the P-TSST is applied.

Directly before and after the stress induction as well as between all later tasks, cortisol measurements were collected using Salivette sampling devices (Sastedt, Nümbrecht, Germany). With each cortisol assessment, we also assessed the mood of the participants (see Fig. 1). For that, we used the Subjective Experiences Rating Scale (SERS^40^) as well as a set of visual analog scales that measure subjective perception of stress (VAS^41^).

### Endocrinological measurements

Saliva analyses were conducted in the in-house laboratory of the Departments of Genetic Psychology and Cognitive Psychology at Ruhr University Bochum. Saliva was analyzed using a cortisol enzyme‐linked immunosorbent assay (Cortisol Saliva ELISA, IBL, Hamburg, Germany) with intra‐assay coefficients of variance (CV) below 5% and interassay CVs below 15%.

In addition, the enzyme alpha amylase (sAA) was analyzed from the saliva samples for assessing the response of the sympathetic nervous system^42^. A colorimetric test using 2-chloro-4-nitrophenyl-α-maltrotriosoide (CNP-G3) as a substrate reagent was applied to measure sAA concentration^43,44^. Intra- and inter-assay variabilities were below 10%.

### Experimental paradigms

#### Banich–Belger task

The Banich–Belger task measures the information transfer capacity of the corpus callosum by assessing interhemispheric integration^31^. Participants were asked to fixate a cross in the middle of the screen while their head was positioned on a chin rest 50 cm away from the screen. Above the fixation cross, two different letters were presented. Simultaneously, a third letter was presented below the fixation cross, either to the right or the left side. The upper letters were presented at 2.8° of visual angle to the side and 1.4° visual angle above the fixation cross. A lower letter was presented at 1.4° visual angle to the left or right and 1.4° visual angle below the fixation cross. The participants were asked to indicate, whether the lower letter coincides with either of the upper letters (button press up) or not (button press down). The paradigm consists of two conditions: in the less demanding physical-matching condition, all letters are upper case; in the more demanding name-matching condition, the lower letter is a lowercase letter. The letters A, B, E, G, H, Q, R, and T were used as stimuli. Both tasks comprised 256 trials in total divided into 4 blocks, the first two belonging to the physical-matching condition and the last two to the name-matching condition. Participants changed the hand with which they responded between each block.

Each trial started by presentation of a fixation cross for 200 ms followed by a stimulus for 200 ms. The intertrial interval jittered between 500 and 2000 ms duration. Prior to the beginning of the task, participants concluded 14 practice trials with each hand, which were excluded from later analyses. Within each block, half of the trials were match trials in which the lower letter did not coincide with either upper letter and half were across-field matches. Within both types of matches, the bottom letter appeared equally often in the right visual field (RVF) and left visual field (LVF). Each block contained 64 trials. Participants changed response hand after each block. The task took about 20 min to complete.

#### Verbal dichotic listening task

For the verbal dichotic listening task, stimuli consisted of syllable pairs made up of two different consonant–vowel pairs (ba, da, ga, ka, pa, ta). Stimuli were presented for a mean duration of 350 ms and at a volume of 80 dB. After stimulus presentation, participants had to press one of six keys labeled with the six consonant–vowel pairs on a customized reaction pad to indicate the syllable they had perceived best. The inter-stimulus interval was randomized between 500 and 1000 ms. Participants first performed a practice run of 12 trials, which were excluded from later analysis. After that, participants completed four blocks consisting of 36 trials each, in which all 36 possible combinations of syllable pairs in the left and right ear were presented. The total number of trials was 144. Participants changed response hand after each block. This task took about 10 min to complete.

#### Emotional dichotic listen task

For the emotional dichotic listening task, only one syllable (dada) was used, which was spoken in five different emotional intonations (happy, sad, neutral, angry, surprised^45^). Participants had to indicate via button press which emotion they had perceived best. The inter-stimulus interval was randomized between 500 and 1000 ms. After a practice run of 12 trials, which was excluded from later analysis, participants completed four blocks consisting of 25 trials each, in which all 25 possible combinations of emotion pairs in the left and right ear were presented. The total number of trials was 100. Participants changed response hand after each block. Similar to the verbal dichotic listening task, this task took about 10 min to complete.

### Statistical analysis

For all further analyses, we used only reaction times and counts of correct responses. In the Banich–Belger task, responses were classified as correct if participants correctly indicated if the lower letter was identical with one of the upper letters. In the dichotic listening tasks, responses were classified as correct if participants responded to either the left or right stimulus. If participants pressed a button corresponding to a stimulus that was not presented, the trial was excluded from further analysis. For the Banich–Belger task, we calculated mean reaction times and mean number of correct responses for each visual half field in each condition for each participant. For both dichotic listening tasks, we calculated mean reaction times and mean number of correct responses for each side of stimulus presentation for each participant.

We calculated lateralization quotients (LQs) in both dichotic listening tasks for mean reaction times and numbers of correct responses following the formula^33,46^:

$${\text{LQ}} = \left[ {\left( {{\text{right}}{-}{\text{left}}} \right)/\left( {{\text{right}} + {\text{left}}} \right)} \right]*100$$

We calculated the across field advantage (AFA) in the Banich–Belger task by subtracting mean reaction times on across-field trials from mean reaction times on within-field trials for the physical-matching and the name-matching task. We also calculated total AFAs by computing an average across name and physical matching condition.

#### Stress response

To evaluate the effectiveness of the stressor, we computed separate repeated measures ANOVAs with the factors session (stress and control) and the measurement time points (T1–T5) using cortisol, alpha amylase as well as SERS and VAS scores as dependent variable. For all later ANOVAs and regression analysis concerning the three behavioral tasks, we log-transformed cortisol and salivary alpha amylase values as the original values were not normally distributed. For the cortisol and sAA data, we calculated the area under the curve with respect to baseline (AUC_i_) following the method detailed in Pruessner et al.^47^. This measure reflects changes in cortisol levels.

We excluded one participant of whom no cortisol data was available. All following analyses were calculated with the remaining 59 participants.

## Results

### Stress induction

**Figure 2:**
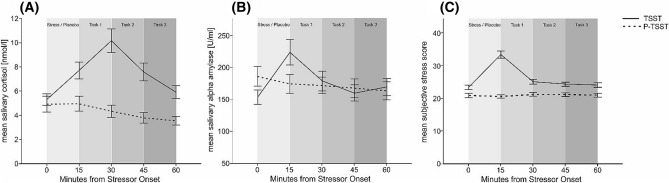
Physiological and subjective stress reactions in the TSST and P-TSST sessions. Error bars represent 1 ± SEM from the mean. (**A**) Mean cortisol responses in relation to measurement time point with non-logarithmized data. (**B**) Mean salivary alpha amylase response in relation to measurement time point. (**C**) Mean subjective stress responses measured by SERS in relation to measurement time point. The first measurement was taken immediately before the TSST or P-TSST preparation period. Task 1–3 refer to the laterality tasks specified in the methods section of the paper.

The comparison between stress and control session (see Fig. 2A) showed a significant main effect of session (F_(1,59)_ = 20.55, *p* < 0.001, η_p_^2^ = 0.26) and measurement time point (F_(4,56)_ = 21.01, *p* < 0.001, η_p_^2^ = 0.26), as well as an interaction effect of both (F_(4,236)_ = 24.37, *p* < 0.001, η_p_^2^ = 0.29). Bonferroni-corrected post-hoc tests revealed a significant increase of salivary cortisol for the second, third and fourth measurement time point compared to the first time point in the stress session (all *p’*s < 0.014, see supplementary table 1 for descriptive data). In the control session, we found significant decreases for measurement time three, four and five compared to baseline measurement (all *p’*s < 0.002).

The analysis for sAA as dependent variable found a significant main effect of measurement time point (F_(4,56)_ = 9.58, *p* < 0.001, η_p_^2^ = 0.14) as well as an interaction effect of session and measurement time point (F_(4,236)_ = 7.77, *p* < 0.001, η_p_^2^ = 0.12, see Fig. 2B). Post-hoc tests revealed that the second measurement time point demonstrated an increase in salivary alpha amylase compared to baseline only in the TSST session (*p* < 0.001, see supplementary table 2 for descriptive data).

The repeated measures ANOVA comparing the SERS and VAS scores during stress and control showed a significant main effect of session (F_(4,59)_ = 51.74, *p* < 0.001, η_p_^2^ = 0.47), main effect of measurement time point (F_(4,236)_ = 38.26, *p* < 0.001, η_p_^2^ = 0.39) as well as an interaction of both (F_(4,236)_ = 47.74, *p* < 0.001, η_p_^2^ = 0.45, see Fig. 2C) for SERS scores. Post-hoc tests revealed that all SERS scores from the second measurement time point on were significantly increased in the stress session (all *p’*s < 0.05, see supplementary table 3 for descriptive data). The same analysis for VAS scores revealed similar results, with a significant main effect of session (F_(4,59)_ = 35.07, *p* < 0.001, η_p_^2^ = 0.37), main effect of measurement time point (F_(4,236)_ = 63.05, *p* < 0.001, η_p_^2^ = 0.52) as well as an interaction of both (F_(4,236)_ = 64.50, *p* < 0.001, η_p_^2^ = 0.52). Post-hoc tests revealed that VAS scores only differed between the two sessions in the second all measurement time points (*p* < 0.001, see supplementary table 4 for descriptive data).

### Banich–Belger task

#### Correct responses

We calculated repeated measures ANOVAs with the factors session (stress vs. control), condition (physical vs. name matching) and visual field (across vs. within) for mean reaction times and number of correct responses. Using correct responses as dependent variable, the ANOVA showed a significant main effect of condition (F_(1,58)_ = 535.02, *p* < 0.001, η_p_^2^ = 0.90), visual field (F_(1,58)_ = 128.48, *p* < 0.001, η_p_^2^ = 0.69) as well as an interaction effect of condition and visual field (F_1,58)_ = 243.49, *p* < 0.001, η_p_^2^ = 0.81, see supplementary Fig. 1a). Bonferroni-corrected post-hoc tests demonstrated a significant increase in correct responses in the name matching condition when performing within-field trials (*p* < 0.001), whereas across-field trials were unaffected (*p* = 0.245). No other main effect of stress session or visual field nor any interactions reached significance (all *p’*s > 0.255).

To identify whether there was indeed no difference between the stress and the control session, we repeated the analysis using a Bayesian repeated measures ANOVA comparing to the null model fit reporting the BF_M_. The BF_M_ reflects the changes from prior odds (no model assumption) to the posterior odds under a certain model in the ANOVA. For t-tests and correlation analyses, we used the BF_10_ factor instead. For both measures, a value of greater than 1 indicates evidence in favor of a hypothesis whereas values lower than 1 indicate that there is evidence against the hypothesis.

We found strong evidence in favor of the alternative hypothesis for the model containing the main effects and interaction effect of condition and visual field (BF_M_ = 94.23). No model containing the factor session provided more than anecdotal evidence for the alternative hypothesis (all BF_M_s < 2.35). Instead, all models except one containing main effects of stress, condition and visual field and an interaction of the latter two (BF_M_ = 2.35) provided strong evidence for the null hypothesis (all BF_M_s < 0.42).

#### Reaction times

We repeated the above-mentioned ANOVA using reaction times as dependent variable. The ANOVA showed a significant main effect of condition (F_(1,58)_ = 8.74, *p* = 0.004, η_p_^2^ = 0.13, see supplementary Fig. 1b). No other main effects or interactions reached significance (all *p’*s > 0.125).

Using a Bayesian repeated measures ANOVA comparing to the null model fit, we found strong evidence in favor of the alternative hypothesis for the model containing the main effect of condition (BF_M_ = 36.71). No model containing the factor stress provided more than anecdotal evidence for the alternative hypothesis (all BF_M_s < 1.68). Instead, all models except one containing main effects of stress and condition (BF_M_ = 1.68) provided strong evidence for the null hypothesis (all BF_M_s < 0.33).

#### Across field advantage

We calculated a dependent sample t-test between AFAs in stress and control sessions for total AFAs. There was no significant difference between the stress and control session (t_(58)_ = 0.20, *p* = 0.845; see supplementary Fig. 2). The same analysis using a Bayesian t-test revealed strong evidence in factor of the null hypothesis (BF_10_ = 0.145).

In a next step, we used Pearson correlations as well as Bayesian correlations to identify whether cortisol, sAA or the subjective stress (SERS) were associated with AFAs.

**Figure 3:**
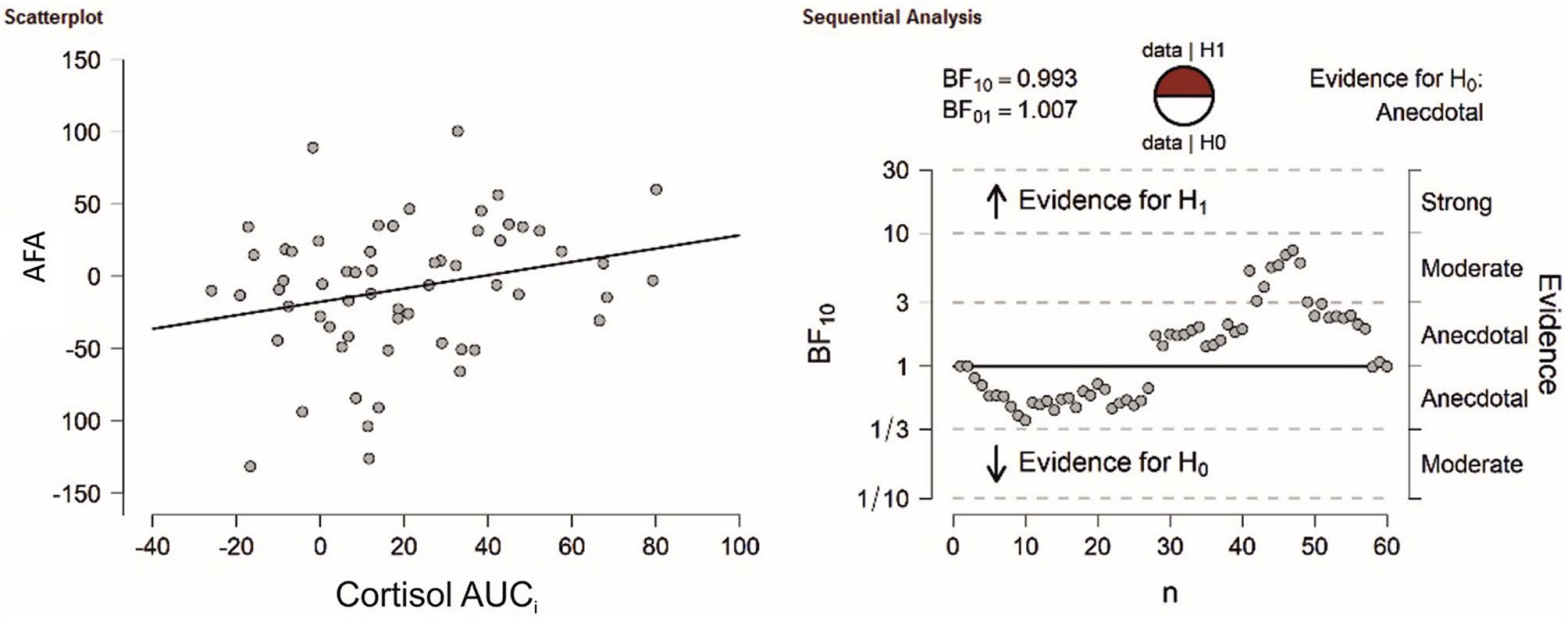
(Left) Correlation analysis between total AFAs and cortisol AUC_i_ in the TSST session. (Right) Bayesian correlation pair analysis for the association between total AFAs and cortisol AUC_i_ across the entire sample. In the top left, the BF10 and BF01 values are presented for which a value of > 1 indicates evidence in favor of the alternative (H1) or null hypothesis (H0), respectively. In the top center, the fraction of data supporting the H1 and H0 is shown. The graph below indicates how evidence in favor of the H1 and H0 develops for each individual participant in the sample. Overall, there was anecdotal evidence in favor of the H0.

For cortisol, there was a trend towards a significant correlation between cortisol and AFAs in the stress session (r_(59)_ = 0.25, *p* = 0.060, see Fig. 3) and no significant association in the control session (r_(59)_ = 0.03, *p* = 0.815). There was anecdotal evidence in favor of the null hypothesis for the stress session (BF_10_ = 0.92) and strong evidence in favor of the null hypothesis for the control session (BF_10_ = 0.17).

For sAA, we found a significant correlation with the AFAs in the stress session (r_(59)_ = 0.28, *p* = 0.032, BF_10_ = 1.55), but not in the control session (r_(59)_ = 0.13, *p* = 0.667, BF_10_ = 0.18) with higher AFAs being associated with higher sAA. There was anecdotal evidence in favor of the alternative hypothesis for the stress session and strong evidence in favor of the null hypothesis for the control session.

For SERS scores, we found no significant association correlation with the AFAs in the stress session (r_(59)_ = 0.07, *p* = 0.578) and the control session (r_(59)_ =  − 0.04, *p* = 0.754). There was strong evidence in favor of the null hypothesis for the stress session (BF_10_ = 0.19) as well as for the control session (BF_10_ = 0.17).

As earlier research has shown, that cortisol and sAA interact influencing memory consolidation^48^, we also chose to investigate the interaction effects of cortisol and sAA on interhemispheric integration. Therefore, we performed a multiple linear regression with log-transformed cortisol and sAA values as predictors for AFAs of reaction times. The model reached overall significance (R^2^ = 0.13, F_(2,56)_ = 4.03, *p* = 0.023) indicating that higher cortisol and sAA values predict higher across-field advantages.

### Verbal dichotic listening task

#### Correct responses

In the verbal dichotic listening task, participants showed a positive LQ for number of correct responses indicating more perceived syllables on the right ear (see supplementary Fig. 3A). The repeated measures ANOVA with the factors session and side revealed only a significant main effect of side (F_(1,58)_ = 64.56, *p* < 0.001, η_p_^2^ = 0.53, see supplementary table 7 for descriptive data) with more correct responses being reported on the right ear. Using a Bayesian repeated measures ANOVA comparing to the null model fit, we found strong evidence in favor of the alternative hypothesis for the model containing the main effect of side (BF_M_ = 23.56). All models containing the factor session provided substantial evidence in favor of the null hypothesis (all BF_M_s < 0.56).

**Figure 4:**
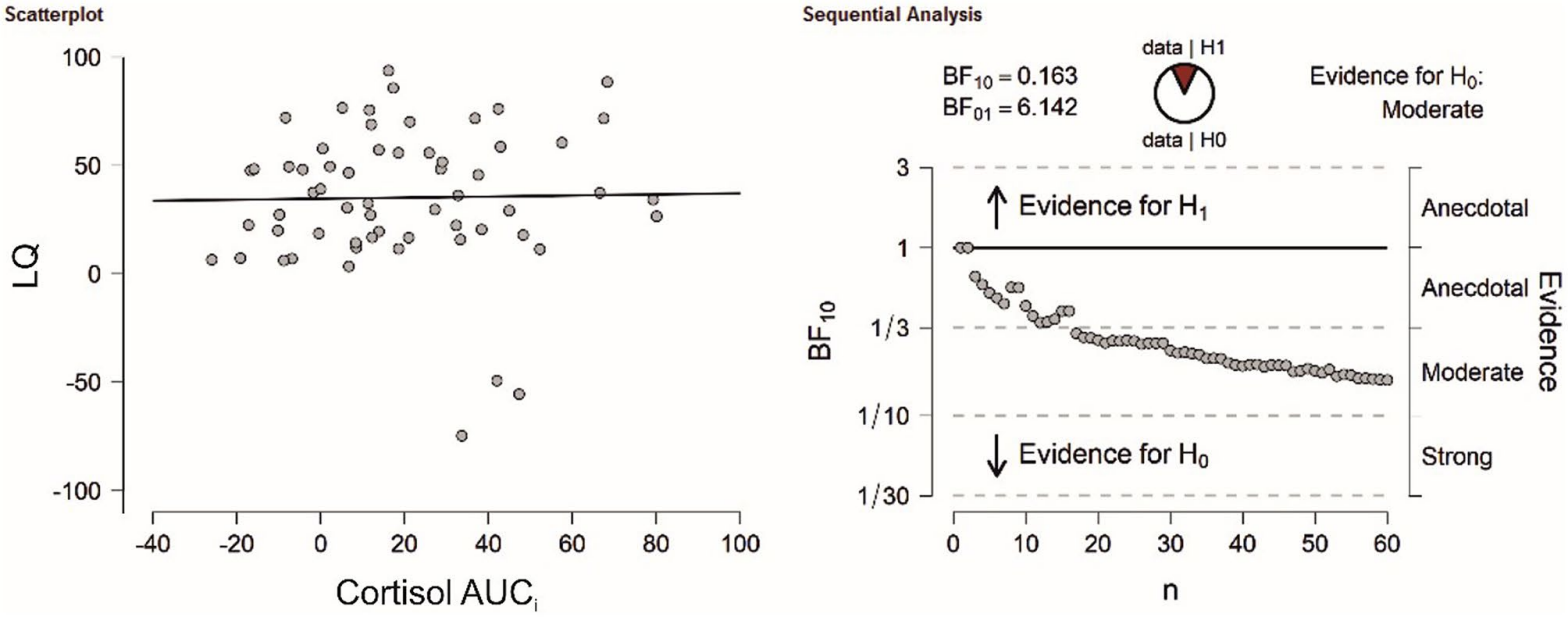
(Left) Correlation analysis between LQs of correct responses in the verbal dichotic listening task and cortisol AUC_i_. in the TSST session. (Right) Bayesian correlation pair analysis for the association between LQs of correct responses and cortisol AUC_i_ across the entire sample. Overall, there was moderate evidence in favor of the H0.

The dependent sample t-test for number of correct responses comparing the stress and control session did not reach significance (t(58) = 0.01, *p* = 0.993, see supplementary Fig. 5A). These results were supported by Bayesian t-tests revealing strong evidence in factor of the null hypothesis for correct responses (BF_10_ = 0.14). There was no significant association with LQs for cortisol (r = 0.02, *p* = 0.884, see Fig. 4), sAA (r = 0.05, *p* = 0.709) or SERS scores (r =  − 0.22, *p* = 0.089) in the stress session. Bayesian correlation analyses indicated in favor of the null hypothesis for all variables (cortisol: BF_10_ = 0.16; sAA: BF_10_ = 0.20; SERS score: BF_10_ = 0.66). For the control session, the results were comparable as no association between correct responses LQs and cortisol (r =  − 0.01, *p* = 0.964), sAA (r =  − 0.26, *p* = 0.848) and SERS scores (r = 0.09, *p* = 0.485) could be detected. As before, Bayesian correlation analyses indicated in favor of the null hypothesis for all variables (cortisol: BF_10_ = 0.16; sAA: BF_10_ = 0.17; SERS score: BF_10_ = 0.20).

#### Reaction times

Participants showed a negative LQ for reaction times indicating faster reactions to stimuli presented on the right ear (see supplementary Fig. 5B). The repeated measures ANOVA with the factors stress and side for mean reaction times revealed only a significant main effect of side (F_(1,58)_ = 16.38, *p* < 0.001, η_p_^2^ = 0.22; see supplementary table 6 for descriptive data) with faster reaction times for stimuli presented on the right ear as well. Using a Bayesian repeated measures ANOVA comparing to the null model fit, we found strong evidence in favor of the alternative hypothesis for the model containing the main effect of side (BF_M_ = 23.52). All models containing the factor stress provided substantial evidence in favor of the null hypothesis (all BF_M_s < 0.54).

**Figure 5:**
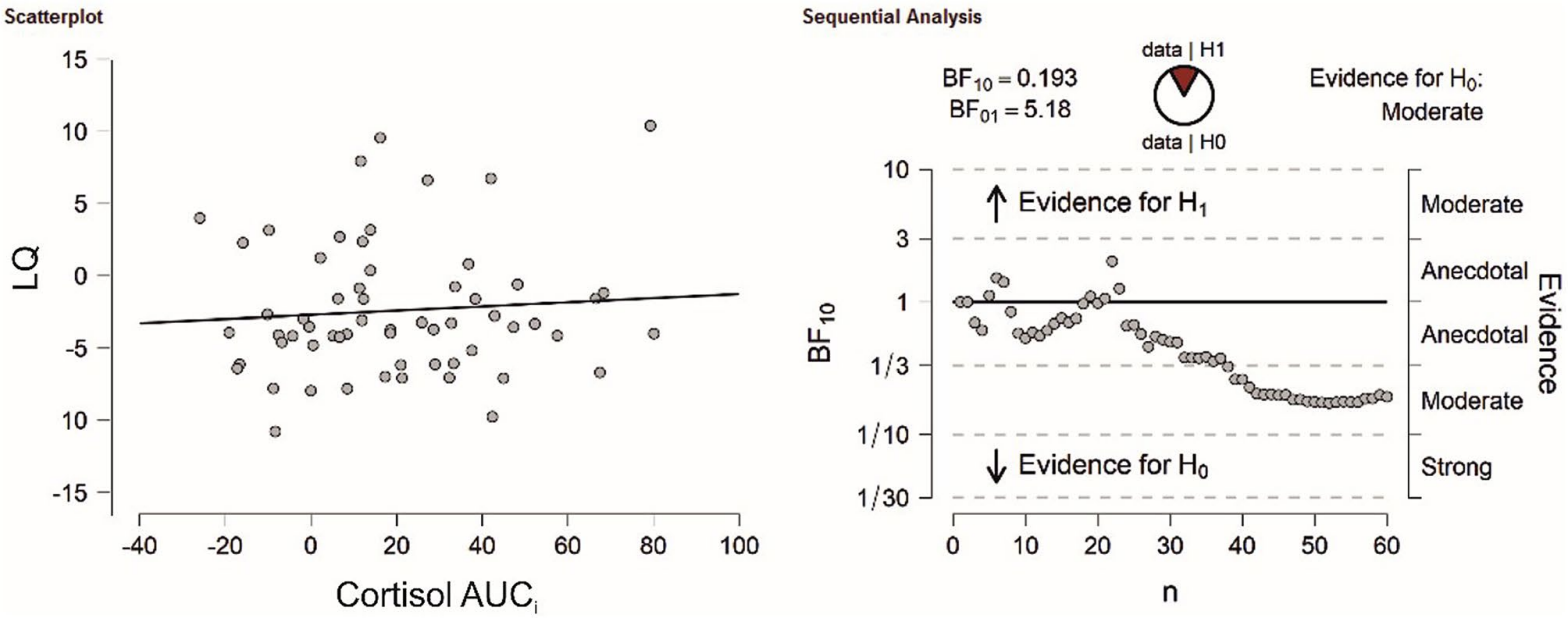
(Left) Correlation analysis between LQs of reaction times in the verbal dichotic listening task and cortisol AUC_i_. in the TSST session. (Right) Bayesian correlation pair analysis for the association between LQs of reaction times and cortisol AUC_i_ across the entire sample. Overall, there was moderate evidence in favor of the H0.

The dependent sample t-test for the LQs of mean reaction times (t_(58)_ = 0.20, *p* = 0.838) comparing the stress and control session did not reach significance. These results were supported by Bayesian t-tests revealing strong evidence in factor of the null hypothesis for reaction times (BF_10_ = 0.15). We then investigated whether individual cortisol, sAA and subjective stress levels were associated with LQs in the verbal dichotic listening task. For reaction times, there was no significant association with LQS for cortisol (r = 0.08, *p* = 0.543, see Fig. 5), sAA (r =  − 0.105, *p* = 0.429) or SERS scores (r = 0.19, *p* = 0.147) in the stress session. Bayesian correlation analyses were in favor of the null hypothesis for all variables (cortisol: BF_10_ = 0.18; sAA: BF_10_ = 0.22; SERS score: BF_10_ = 0.45). For the control session, the results were comparable as no association between reaction times LQs and cortisol (r = 0.16, *p* = 0.220), sAA (r = 0.08, *p* = 0.530) and SERS scores (r =  − 0.13, *p* = 0.317) could be detected. Again, Bayesian correlation analyses indicated in favor of the null hypothesis for all variables (cortisol: BF_10_ = 0.17; sAA: BF_10_ = 0.19; SERS score: BF_10_ = 0.26).

### Emotional dichotic listening task

#### Correct responses

In the emotional dichotic listening task, participants on average showed a negative LQ for number of correct responses indicating more perceived syllables on the left ear (see supplementary table 9 for descriptive data). The repeated measures ANOVA with the factors stress and side revealed only a significant main effect of side (F_(1,58)_ = 21.95, *p* < 0.001, η_p_^2^ = 0.28, see supplementary Fig. 4A) with more correct responses being reported on the left ear. Using a Bayesian repeated measures ANOVA comparing to the null model fit, we found strong evidence in favor of the alternative hypothesis for the model containing the main effect of side (BF_M_ = 23.11). All models containing the factor session provided substantial evidence in favor of the null hypothesis (all BF_M_s < 0.57).

**Figure 6:**
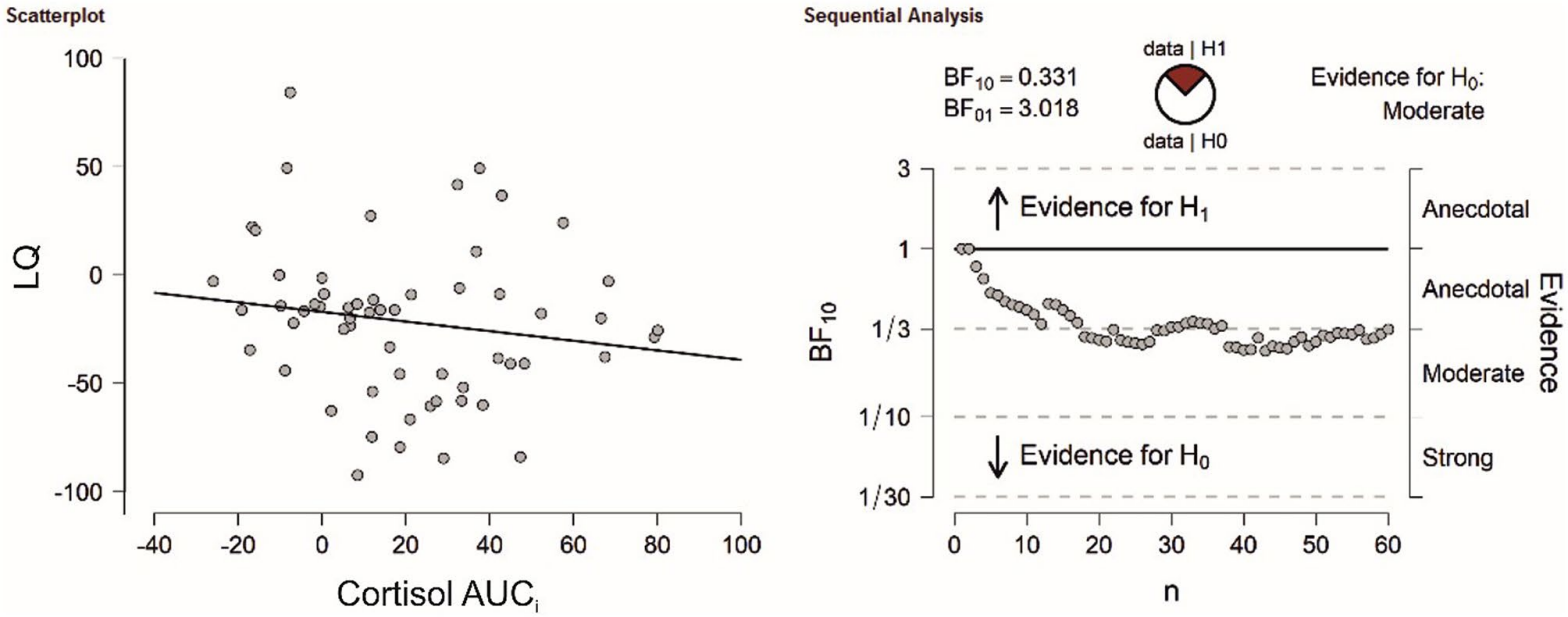
(Left) Correlation analysis between LQs of correct responses in the emotional dichotic listening task and cortisol AUC_i_ in the TSST session. (Right) Bayesian correlation pair analysis for the association between LQs of correct responses and cortisol AUC_i_ across the entire sample. Overall, there was moderate evidence in favor of the H0.

The dependent sample t-test for number of correct responses comparing the stress and control session did not reach significance (t_(58)_ = 0.14, *p* = 0.890). These results were supported by Bayesian t-tests revealing strong evidence in factor of the null hypothesis for correct responses (BF_10_ = 0.14). There was no significant correlation with LQS for cortisol (r =  − 0.16, *p* = 0.224, see Fig. 6), sAA (r =  − 0.11, *p* = 0.407) or SERS scores (r = 0.08, *p* = 0.531) in the stress session. Bayesian correlation analyses indicated in favor of the null hypothesis for all variables (cortisol: BF_10_ = 0.31; sAA: BF_10_ = 0.23; SERS score: BF_10_ = 0.20). For the control session, the results were comparable as no association between correct responses LQs and cortisol (r =  − 0.13, *p* = 0.334), sAA (r =  − 0.110, *p* = 0.407) and SERS scores (r =  − 0.05, *p* = 0.714) could be detected. As before, Bayesian correlation analyses indicated in favor of the null hypothesis for all variables (cortisol: BF_10_ = 0.27; sAA: BF_10_ = 0.21; SERS score: BF_10_ = 0.17).

#### Reaction times

Participants showed a positive LQ for reaction times indicating faster reactions to stimuli presented on the left ear (see supplementary Fig. 4B). The repeated measures ANOVA with the factors stress and side for mean reaction times revealed only a significant main effect of side (F_(1,58)_ = 21.15, *p* < 0.001, η_p_^2^ = 0.27; see supplementary table 8 for descriptive data) with faster reaction times for stimuli presented on the left ear as well. Using a Bayesian repeated measures ANOVA comparing to the null model fit, we found strong evidence in favor of the alternative hypothesis for the model containing the main effect of side (BF_M_ = 19.01). All models containing the factor stress provided substantial evidence in favor of the null hypothesis (all BF_M_s < 0.65).

**Figure 7:**
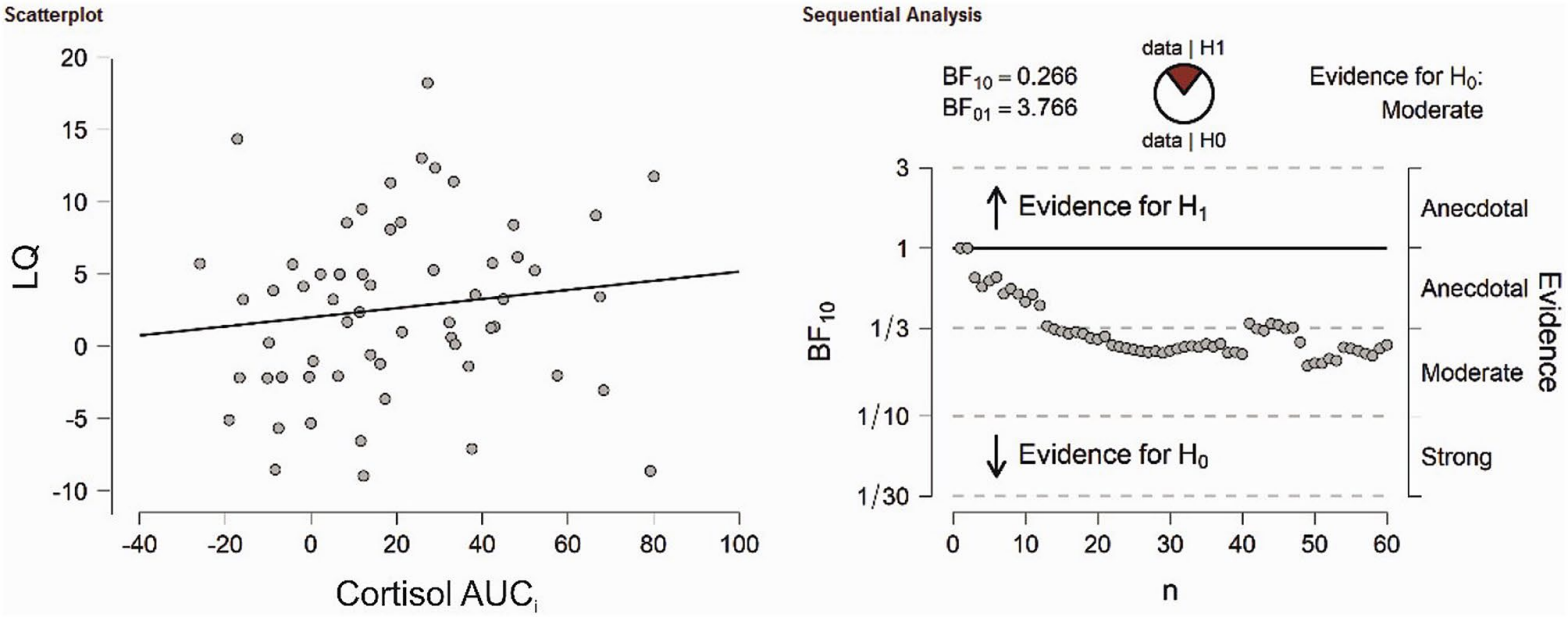
(Left) Correlation analysis between LQs of reaction times in the emotional dichotic listening task and cortisol AUC_i_ in the TSST session. (Right) Bayesian correlation pair analysis for the association between LQs of reaction times and cortisol AUC_i_ across the entire sample. Overall, there was moderate evidence in favor of the H0.

The dependent sample t-test for the LQs of mean reaction times comparing the stress and control session did not reach significance (t_(58)_ = 0.68, *p* = 0.497). These results were supported by Bayesian t-tests revealing strong evidence in factor of the null hypothesis for reaction times (BF_10_ = 0.18). We then investigated whether individual cortisol, sAA and subjective stress were associated with LQs in the verbal dichotic listening task. For reaction times, there was no significant correlation with LQS for cortisol (r = 0.13, *p* = 0.312, see Fig. 7) or SERS scores (r = 0.09, *p* = 0.490) in the stress session. There was however a trend between LQs and sAA (r =  − 256, *p* = 0.051). Bayesian correlation analyses for the association between cortisol and SERS scores indicated in favor of the null hypothesis (cortisol: BF_10_ = 0.27; SERS score: BF_10_ = 0.20). There was anecdotal evidence in favor of the alternative hypothesis for sAA (BF_10_ = 1.05). For the control session, we found no association between correct responses LQs and cortisol (r = 0.03, *p* = 0.838), sAA (r =  − 0.43, *p* = 0.749), and SERS scores (r =  − 0.07, *p* = 0.613). Bayesian correlation analyses for the association indicated in favor of the null hypothesis for all variables (cortisol: BF_10_ = 0.16; sAA: BF_10_ = 0.25; SERS score: BF_10_ = 0.18).

## Discussion

The aim of the current study was to investigate the influence of acute stress on FHAs. To this end, participants performed three tasks measuring FHAs in one session with and one without stress induction. For this, we used the TSST, a stress induction paradigm that has been used extensively in stress research^49^ and comprises elements of social evaluation and performance tasks, both known to induce a cortisol response^39^. Like several previous studies, we could observe a reliable increase in cortisol and sAA as well as subjective stress in the TSST compared to the P-TSST session^50^. In the P-TSST session, we could replicate the results found widely in the literature in both dichotic listening as well as the Banich–Belger task (see supplementary results).

For the verbal dichotic listening task, we replicated the right-ear advantage for reaction times and numbers of reported syllables indicating leftward language lateralization^51–53^. For our analysis of same-voiced syllable pairs (see supplementary), we found more reported stimuli for syllable pairs with short voice onset time. For the emotional dichotic listening, a clear left-ear advantage was evident representing a right-hemispheric dominance for processing of emotional stimuli^30,54,55^. In the Banich–Belger task, participants were more accurate on across-field trials compared to within-field trials in the more complex name-matching condition demonstrating an across-field-advantage (AFA) as expected^31,56,57^.

In contrast to our initial hypothesis, we could not find statistically significant differences in accuracy or reaction times between the stress and the control session in all three paradigms. Moreover, there were no significant correlations between dichotic listening LQs in both tasks and any stress measurement. The analysis for same-voiced syllable pairs also did not reveal an influence of stress (see supplementary). These findings were confirmed using both Bayesian and frequentist statistics. Contrary to the assumptions of the psychoneuroendocrine model, stress related cortisol increase largely did not influence FHAs. In contrast, there was a significant association between sAA levels and AFAs in the stress session. Moreover, Bayesian analyses indicated anecdotal evidence for an association between the across-field advantage for reaction times and number of correct responses in the Banich–Belger task and increases in cortisol. This indicates weak evidence that stronger increases in cortisol and salivary alpha amylase were accompanied by higher AFAs. As the AFAs benefit from bihemispheric integration, it is conceivable that both, HPA axis activity and sympathetic activity, enhances general information transfer between the hemispheres. These results are in line with earlier work by Compton and Mintzer^58^, who found that evaluative stress lead to a greater advantage of interhemispheric integration of information across the corpus callosum. A potential mechanism supporting this association could relate to the function of the corpus callosum^59^. FHAs originate through homotopic inhibition by the contralateral hemisphere^60^: glutamatergic callosal fibers project onto contralateral inhibitory interneurons leading to an inhibition of the non-dominant hemisphere^61^. As the AFAs benefit from integration across both hemispheres, increased interhemispheric coupling via inhibition would rather lead to decreased AFAs^62^. This is however not necessarily in opposition to a possible influence of cortisol on interhemispheric integration as measures with the Banich–Belger task, as the extent to which interhemispheric inhibition and interhemispheric integration share the same transcallosal mechanisms is not clear^63^. The effect of sympathetic activations indicated by salivary alpha amylase could be mediated by similar mechanisms. As the effect of sAA reached significance and the cortisol effect was at trend level, it is conceivable that the influence of acute stress on interhemispheric integration might be among other things mediated by sympathetic activation. This is supported by the results of the multiple linear regression which showed an interaction effect between cortisol and sAA on interhemispheric integration.

There is ample evidence that sex hormones like progesterone levels influence FHAs by interacting with glutamatergic and GABAergic transcallosal signaling leading to a decoupling of the two hemispheres^36^. As hemispheric asymmetries are partially dependent on interhemispheric inhibition, high levels of cycling phase dependent female sex hormones like progesterone reduce this inhibition by decreasing callosal synaptic efficiency. Similar effects have been reported for estradiol^64,65^ and testosterone^66^.

In contrast to progesterone, cortisol enhances glutamatergic transmission and should lead to enhanced FHAs^25^. Thus, an increase in FHAs would be expected. However, our results indicate that at least on a behavioral level, cortisol did not exert any influence on corpus callosum function. This is contrary to the results of Brüne et al.^18^, who found that acute stress induces asymmetrical processing in an emotional dot probe tasks. However, in their study, Brüne et al.^18^ utilized a paradigm that is not classically used to measure emotional lateralization. Rather, the focus of their study laid on emotional attention, which was modified by stress. This indicates that attentional processes might be asymmetrically affected by stress rather than the emotional lateralization itself. As we found stronger effects of sAA on interhemispheric integration than of cortisol, future studies investigating this relation need to include a measurement of noradrenergic activity. Many studies investigating the influence of sex hormones on FHAs also typically use tasks measuring visual spatial attention^65^, so these asymmetries might be especially sensitive to the influence of steroid hormones of corpus callosum function. In our study, we focused on emotion and language lateralization. Thus, future studies investigating hemispheric asymmetries under stress with other paradigms than dichotic listening are needed. Our results are not in accordance with a recent model in which stress leads to functional equalization of the right and left hemisphere^67^. This equalization would reveal itself on a behavioral level as decreased FHAs, which we could not find in our study. However, the authors also propose that the brain possesses a high capacity for redistribution of hemispheric performance under stress. This notion would be supported by the fact that stress leads to a better interhemispheric integration in our study.

In the context of stress, more attention needs to be paid to the emotional dichotic listening task as stressful social situations lead to increased sensitivity to emotional stimuli^8,68^. It has also been shown that the effects of cortisol on memory and other cognitive processes interact with emotional arousal and the concomitant increase in catecholamines^48,69–71^. Thus, the processing of emotional stimuli in the emotional dichotic listening task might be differentially affected by cortisol and subjective stress. If the negative affect resulting from the stress induction would prime the right hemisphere selectively as it dominantly processes emotion^21,22^, changes in emotional lateralization might thus be affected by other stress mediators than cortisol.

While we did not find any changes in asymmetry due to acute stress, there is evidence that early life stress and chronic elevation of the HPA-axis are associated with changes structural and functional hemispheric asymmetries in several mental and neurodevelopmental disorders. Similarly to the Fetal Origins of Mental Health hypothesis by O’Donnell and Meaney^72^, intrauterine factors which program neural systems underlying cognitive-emotional function could not only affect the development of mental and neurodevelopmental disorders but also the development of hemispheric asymmetries adversely through epigenetic regulation. For example, there is evidence that maternal stress plays a role in the development of handedness^73^. Typically, in mental and neurodevelopmental disorder, patients show increases in cortisol and decreases in asymmetries. One could speculate that the influence of acute stress on laterality might be influenced by long-term cortisol exposure in the brain. In a recent study, Mundorf et al.^74^ exposed newborn rats to chronic stress via separation and isolated housing. Chronic stress exposure during early life lead to stronger leftward asymmetry in turning behavior. This demonstrates an association between laterality and stress on a chronic level. Thus, future studies should assess possible early life stressors as influencing factors.

### Limitations and outlook on future studies

A limitation of the current study is the possibility of confounding hormonal influences in the female participants. As the stage of the menstrual cycle is known to interact with FHAs, we only tested women in the luteal phase^36^. Here, we relied on self-report of participants to estimate the cycle phase. As the cycle phase also influences cortisol reactivity to stress induction^75^, measuring levels of reproductive hormones in future studies would be interesting to test for possible interactions with cortisol.

The present study used a purely behavioral approach. The tasks employed in the present study strongly rely on interhemispheric processing, thus possible effects of cortisol on these tasks results can be expected to reflect changes in callosal information transfer. However, it would be interesting to further investigate into the underlying processes to look for an influence of cortisol on asymmetries on a neuronal level. Since it is not clear how cortisol interacts with information transmission properties of the CC, using EEG recordings could illuminate on transhemispheric processing differences under stress. Direct measures of neurophysiological processes would advance our understanding of underlying neural mechanisms, hence using techniques like fMRI might be helpful in investigating this association. Furthermore, it cannot be excluded entirely that stress had a selective effect on the right hemisphere as there is evidence that the HPA-axis is dominantly controlled by the right hemisphere^24^. It would be interesting to see whether the administration of cortisol directly would lead to different results as in our study instead of inducing a cortisol reaction via the TSST.

Finally, the potential absence of significant differences between the TSST and P-TSST session might result from the type of stressor. In our study, social stress was used for stress induction. It could be speculated that social stress is especially impactful on social tasks. The effects of stress therefore could be modality specific. If for example an auditory stressor were to be used, it could have stronger effects on the emotional or verbal dichotic listening tasks.

### Conclusion

In conclusion, the current study could not show a relation between cortisol and hemispheric asymmetries under acute stress. In contrast, there was evidence for an influence of cortisol and sympathetic activation indicated by salivary alpha amylase changes on AFAs in the Banich–Belger task. This indicates that acute stress does seem to affect interhemispheric integration of information. Thus, future research on stress and FHAs should investigate if cortisol has an influence on information transfer across the corpus callosum on a neural level. Studies utilizing hydrocortisone and EEG would be suited for this undertaking.

## Supplementary information

Supplementary information.
