# Supplementary material for: Dichotic listening performance and interhemispheric integration after stress exposure
Source: Sci Rep. 2020 Nov 30;10:20804. doi: 10.1038/s41598-020-77708-5 (PMC7705688; doi:10.1038/s41598-020-77708-5)
Supplement: Supplementary file 1 — Supplementary information. [file 41598_2020_77708_MOESM1_ESM.docx]

Dichotic listening performance and interhemispheric integration after stress exposure

Gesa Berretz^1*^, Julian Packheiser^1^, Oliver T. Wolf^2^, Sebastian Ocklenburg^1, 3^

^1^: Department of Biopsychology, Institute of Cognitive Neuroscience, Faculty of Psychology, Ruhr University Bochum, Bochum, Germany

^2^: Department of Cognitive Psychology, Institute of Cognitive Neuroscience, Faculty of Psychology, Ruhr University Bochum, Bochum, Germany

^3^: Department of Psychology, University of Duisburg-Essen, Essen, Germany

*Corresponding Author: Gesa Berretz

Telephone Number: +49 234 32 21453

Fax Number: +49 234 32 14377

E-Mail: Gesa.Berretz@rub.de

Supplementary Table 1: Cortisol (not logarithmized)

| Stress | Measurement time point 1 | Measurement time point 2 | Measurement time point 3 | Measurement time point 4 | Measurement time point 5 |
| --- | --- | --- | --- | --- | --- |
| TSST | 5.42 ± 0.51 | 7.69 ± 0.77 | 9.70 ± 0.96 | 6.93 ± 0.59 | 5.48 ± 0.47 |
| P-TSST | 4.30 ± 0.37 | 4.35 ± 0.37 | 3.78 ± 0.30 | 3.32 ± 0.27 | 3.12 ± 0.24 |

Supplementary Table 2: Alpha Amylase (not logarithmized)

| Stress | Measurement time point 1 | Measurement time point 2 | Measurement time point 3 | Measurement time point 4 | Measurement time point 5 |
| --- | --- | --- | --- | --- | --- |
| TSST | 155.64 ± 12.01 | 221.12 ± 20.47 | 178.58 ± 16.45 | 163.49 ± 13.75 | 174.54 ± 14.19 |
| P-TSST | 189.11 ± 16.31 | 173.93 ± 15.14 | 171.43 ± 13.42 | 168.61 ± 15.82 | 162.60 ± 14.86 |

Supplementary Table 3: SERS

| Stress | Measurement time point 1 | Measurement time point 2 | Measurement time point 3 | Measurement time point 4 | Measurement time point 5 |
| --- | --- | --- | --- | --- | --- |
| TSST | 21.67 ± 5.65 | 31.68 ± 8.69 | 23.40 ± 5.59 | 22.65 ± 5.13 | 22.37 ± 5.50 |
| P-TSST | 20.87 ± 5.12 | 20.55 ± 3.70 | 21.21 ± 3.94 | 21.15 ± 4.44 | 21.02 ± 4.63 |

Supplementary Table 4: VAS

| Stress | Measurement time point 1 | Measurement time point 2 | Measurement time point 3 | Measurement time point 4 | Measurement time point 5 |
| --- | --- | --- | --- | --- | --- |
| TSST | 279.25 ± 74.18 | 559.60 ± 142.49 | 315.57 ± 105.51 | 319.55 ± 95.53 | 331.86 ± 115.14 |
| P-TSST | 267.08 ± 92.40 | 293.08 ± 80.05 | 313.37 ± 101.87 | 314.78 ± 90.61 | 326.73 ± 104.87 |

Supplementary Table 5: AFA

| Stress | Physical | Name |
| --- | --- | --- |
| TSST | -19.00 ± 6.09 | 2.20 ± 12.73 |
| P-TSST | -10.15 ± 6.91 | 4.61 ± 16.65 |

Supplementary Table 6: vDLT reaction times

|  | Left ear | Right ear |
| --- | --- | --- |
| TSST | 139.73 ± 23.89 | 132.52 ± 20.12 |
| P-TSST | 139.99 ± 22.26 | 132.60 ± 18.42 |

Supplementary Table 7: vDLT correct answers

|  | Left ear | Right ear |
| --- | --- | --- |
| TSST | 28.87 ± 15.35 | 59.32 ± 16.18 |
| P-TSST | 28.91 ± 15.60 | 58.77 ± 17.76 |

Supplementary Table 8: eDLT reaction times

|  | Left ear | Right ear |
| --- | --- | --- |
| TSST | 163.47 ± 37.18 | 171.48 ± 37.33 |
| P-TSST | 163.68 ± 32.54 | 173.55 ± 32.93 |

Supplementary Table 9: eDLT correct responses

|  | Left ear | Right ear |
| --- | --- | --- |
| TSST | 41.32 ± 1.95 | 27.70 ± 1.82 |
| P-TSST | 41.59 ± 2.13 | 27.83 ± 1.91 |

Supplementary Results

Proof of principle for Banich-Belger task

To determine, whether the Banich-Belger task worked as predicted by the literature, we calculated a repeated measures ANOVA for numbers of correct responses within the P-TSST session with the factors condition (physical vs name matching) and visual field (across vs. within). There was a significant main effect of task (F_(1,52)_ = 231.64, p<.001, η_p_^2^ = .82) and visual field (F_(1,52)_ = 127.5, p<.001, η_p_^2^ = .71) as well as an interaction effect of both (F_(1,52)_ = 364.55, p<.001, η_p_^2^ = .64, see supplementary figure 1). Bonferroni-corrected post hoc tests revealed that the participants reported significantly more correct responses in name matching compared to physical matching on within-field trials (p < .001). No differences were observed when the task was performed on across-field trials (p = .646). We performed the same analysis for reaction times. No comparisons reached significance (all *p’*s > .075).


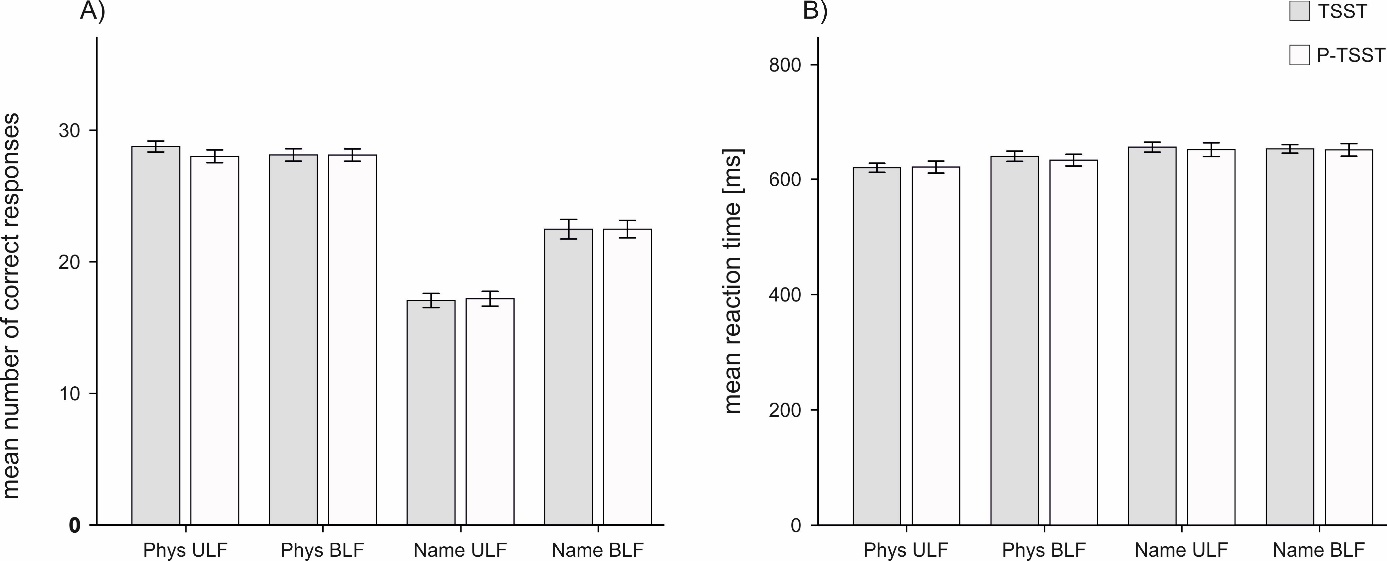


Supplementary figure 1. Mean number of correct responses (A) and mean reaction times (B) for each condition and visual field in the Banich-Belger task. Error bars represent ±1 SEM.

Across-field advantage


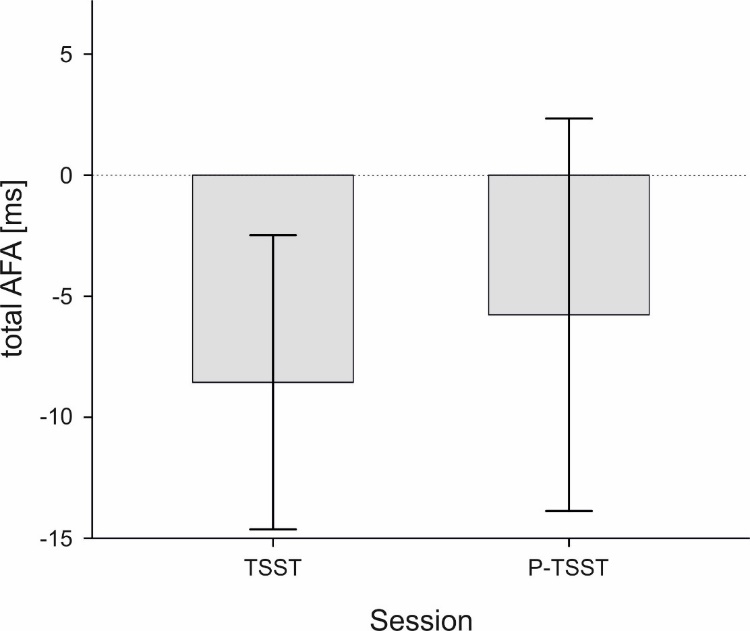


Supplementary figure 2. Total across-field advantage for reaction times in the Banich-Belger task. Error bars represent ±1 SEM.

Proof of principle for Dichotic listening tasks

To see if the verbal dichotic listening task produced a similar pattern as predicted in the literature, we calculated a dependent sample t-test to compare mean reaction times and numbers of responses the stimuli on the right and left side within the P-TSST session. Analyses revealed that participants reported significantly more syllables on the right ear (t_(52)_ = 6.84, p<.001, mean = 58.77 ± 17.76) than on the left ear (mean = 28.91 ± 15.61, see supplementary figure 3A) and reacted faster to stimuli on the right side (t_(52)_ = 4.15, p<.001, mean = 132.60ms ± 18.42) than on the left side (mean = 139.99ms ± 22.26; see supplementary figure 3B).


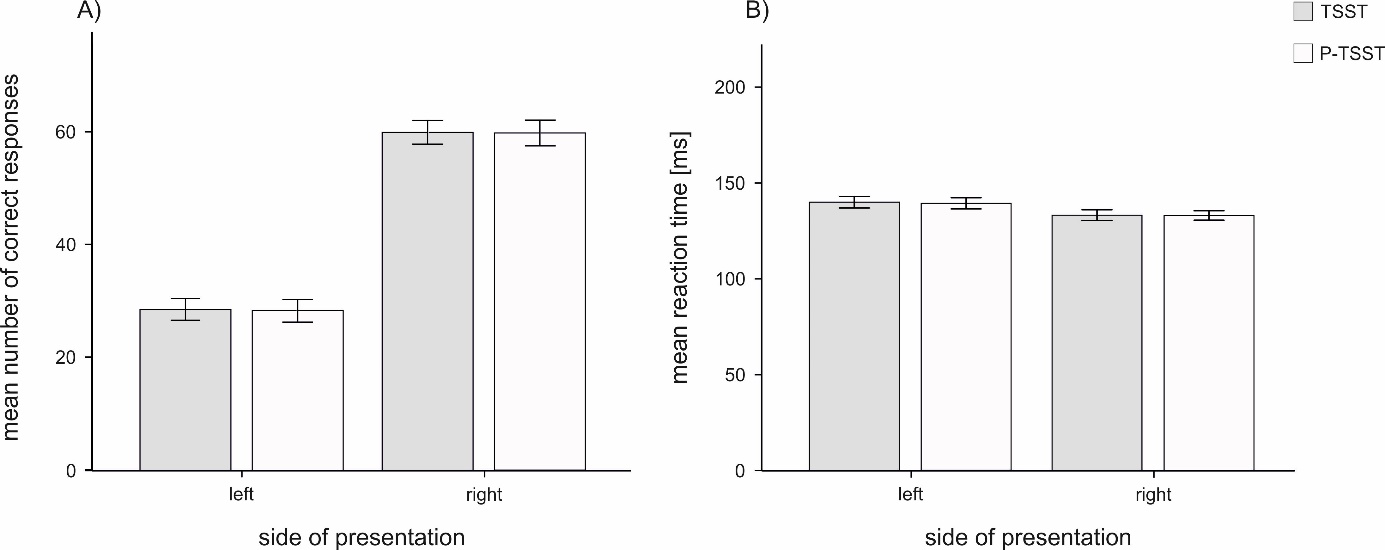


Supplementary figure 3. Mean number of reactions per side (A) and mean reaction times (B). Participants reported more stimuli presented and were faster in responding to stimuli on the right than on the left side indicating a left hemispheric lateralization for language. Error bars represent ±1 SEM.

To see if the emotional dichotic listening task produced a similar pattern as predicted in the literature, we calculated a dependent sample t-test to compare mean reaction times and numbers of responses the stimuli on the right and left side within the P-TSST session. Analyses revealed that participants reported significantly more syllables on the left ear (t_(52)_ = 3.59, p=.001, mean = 41.58 ± 15.47) than on the right ear (mean = 27.83 ± 13.87, see supplementary figure 4A) and reacted faster to stimuli on the left side (t_(52)_ = 3.58, p=.001, mean = 163.86ms ± 32.54) than on the right side (mean = 173.55ms ± 32.93, see supplementary figure 4B).


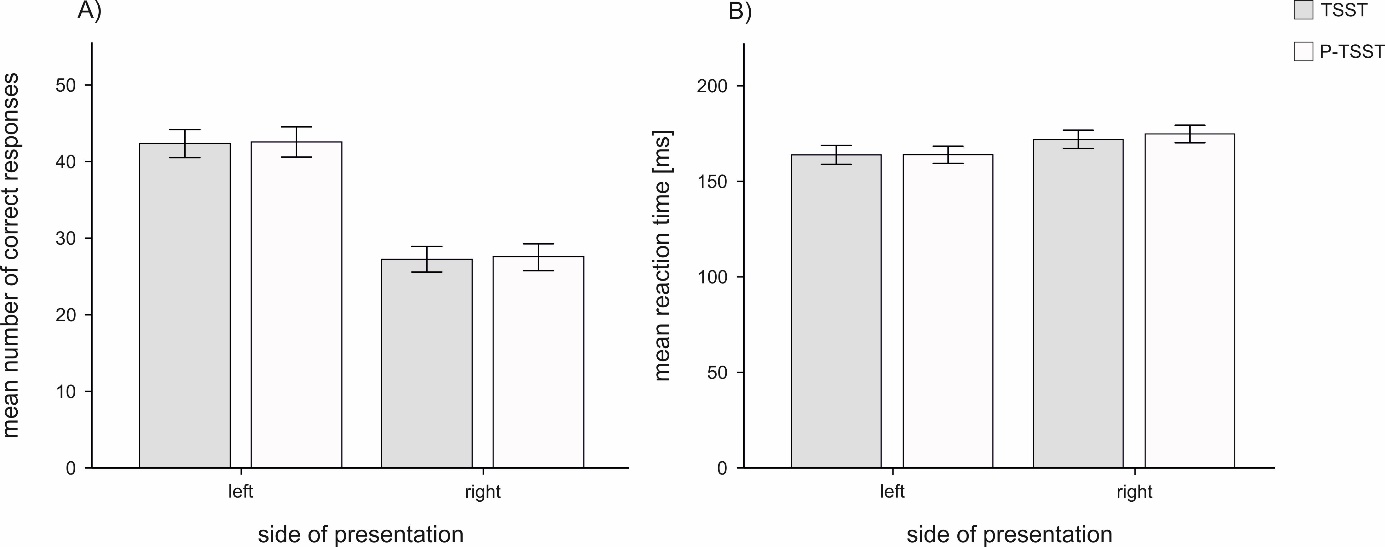


Supplementary figure 4. Mean number of reactions per side (A) and mean reaction times (B). Participants reported more stimuli presented and were faster in responding to stimuli on the left than on the right side indicating a right hemispheric lateralization for emotion processing. Error bars represent ±1 SEM.


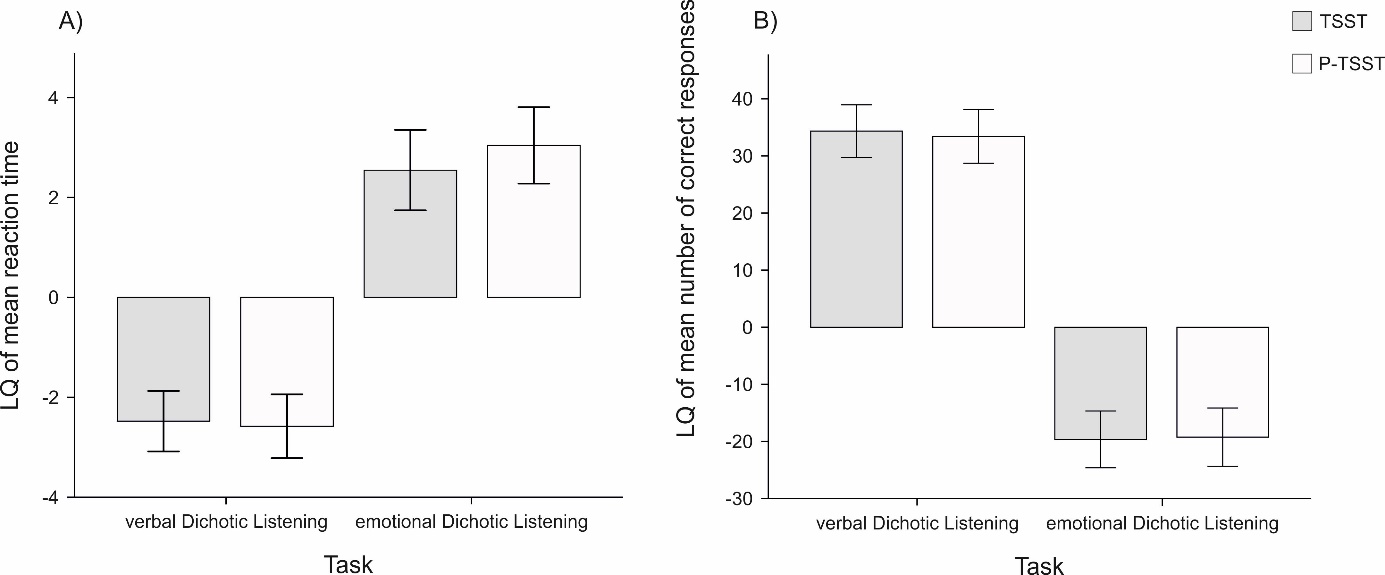


Supplementary figure 5. LQs for the verbal and emotional dichotic listening task for mean reaction times (A) and number of correct responses (B). Error bars represent ±1 SEM.

Analysis of same-voiced syllable pairs in the verbal DLT

In our main analysis, we analyzed same- (both syllables have long or short voice onset times) and mixed-voiced (one syllable has a short voice onset time while the other syllable has a long voice onset time) dichotic stimulus pairs together. However, previous studies have shown that the mixed-voiced pairs likely demand a stronger involvement of top-down cognitive control [1] and can display an altered ear advantage [2]. To identify possible confounding influences of mixed-voiced stimuli on correct responses, we performed a repeated measures ANOVA with the factors session (stress vs. placebo), side (left vs. right) and length (short vs. long) with only same voiced trails. The ANOVA revealed a significant main effect of side (F_(1,58)_ = 64.08, p<.001, η_p_^2^ = .53) and length (F_(1,58)_ = 205.19, p<.001, η_p_^2^ = .78) indicating more correct responses to stimuli presented on the right side as well as short stimuli. No other main effects or interactions reached significance (all *p*s >.098). The dependent sample t-test for LQs of correct responses comparing the stress and control session did not reach significance for short (t_(58)_ = -1.02, *p* = .313) nor long (t_(58)_ = -0.78, *p* = .439) syllables. There was no significant association between LQs of long syllables and cortisol (r = .09, *p* = .503), sAA (r = 0.05, *p* = .727) or SERS scores (r = .076, *p* = .567) in the stress session. For short syllables, there was also no significant association between LQs of long syllables and cortisol (r = 0.02, *p* = .870), sAA (r = 0.07, *p* = .594) or SERS scores (r = .105, *p* = .428) in the stress session.

Additionally, we performed a paired-sample t-test on stimuli presented to the left ear to evaluate, if stress had an effect of stimuli that were integrated across the corpus callosum [3,4]. There was no significant difference between the two sessions (t_(58)_ = .408, *p* = .685).

References

1. Westerhausen, R., Passow, S. & Kompus, K. Reactive cognitive-control processes in free-report consonant-vowel dichotic listening. *Brain and cognition* **83,** 288–296 (2013).

2. Westerhausen, R. A primer on dichotic listening as a paradigm for the assessment of hemispheric asymmetry. *Laterality* **24,** 740–771 (2019).

3. Steinmann, S. *et al.* Conscious auditory perception related to long-range synchrony of gamma oscillations. *NeuroImage* **100,** 435–443 (2014).

4. Westerhausen, R. & Hugdahl, K. The corpus callosum in dichotic listening studies of hemispheric asymmetry: a review of clinical and experimental evidence. *Neuroscience & Biobehavioral Reviews* **32,** 1044–1054 (2008).
